# Increasing SARS-CoV-2 nucleic acid testing capacity during the COVID-19 epidemic in Beijing: experience from a general hospital

**DOI:** 10.1080/22221751.2020.1837016

**Published:** 2020-11-01

**Authors:** Qi Wang, Xiaotao Zhao, Zhihong Yue, Xiaohui Bai, Huiying Rao, Na Han, Tianbing Wang, Hui Wang, Baoguo Jiang

**Affiliations:** a Department of Clinical Laboratory, Peking University People’s Hospital, Beijing, People’s Republic of China; b Department of Clinical Laboratory, Provincial Hospital Affiliated to Shandong University, Jinan, People’s Republic of China; c Office of Academic Research, Peking University People’s Hospital, Beijing, People’s Republic of China; d Trauma Center, Peking University People’s Hospital, Beijing, People’s Republic of China

**Keywords:** SARS-CoV-2, nucleic acid testing, COVID-19, PCR laboratory, testing capacity

## Abstract

Under the ongoing COVID-19 prevention and control measures in China, increasing the laboratory severe acute respiratory syndrome coronavirus 2 (SARS-CoV-2) nucleic acid testing capacity has become the top priority. Since the COVID-19 outbreak in Xinfadi market in Beijing in June 2020, large-scale screening of key populations has been carried out, challenging the nucleic acid testing capabilities of hospital laboratories. Therefore, within 48 hours, Peking University People’s Hospital (PKUPH) transformed a non-nucleic acid testing laboratory into a SARS-CoV-2 nucleic acid testing laboratory. Based on the original structure of the building, we adapted measures to local conditions, sorted out a new testing process, and quickly started testing for COVID-19. The nucleic acid testing process has been optimized, including quality control, personal operating specifications, and the timeliness of the release of LIS results to form closed-loop management. This high-throughput COVID-19 testing optimization process provides a reference model for other countries that are fighting the epidemic.

The spread of coronavirus disease (COVID-19) worldwide has caused considerable economic losses and has affected people’s daily lives [1]. Since Beijing announced a newly confirmed COVID-19 outbreak associated with Xinfadi market on June 11, 2020, the Beijing Municipal Government have rapidly implemented precise testing for the prevention and control of the epidemic. Within just 1 month, the epidemic in Beijing was successfully contained, and there were no increases in the number of newly confirmed cases. Accurate and efficient nucleic acid detection and improvements in nucleic acid detection capacity are essential for epidemic prevention and control [2]. However, many countries and regions have insufficient testing capacities, which makes controlling the COVID-19 epidemic difficult [3]. The WHO recommendations do not give recommendations for the handling of large number of specimens [4]. Our processing summary is to increase the speed, especially when dealing with large samples. From June 11 to July 14, 2020, Beijing performed 11.88 million SARS-CoV-2 nucleic acid tests. More than 50% of people infected with SARS-CoV-2 in Beijing were identified through active screening by nucleic acid testing [5].

On June 20, 2020, Peking University People’s Hospital (PKUPH) urgently reconstructed an out-of-use laboratory on another campus as a SARS-CoV-2 nucleic acid testing laboratory to increase the volume of nucleic acid testing. To perform the required nucleic acid tests, the laboratory required modifications included physical partitioning of staff and equipment, alterations to airflow direction and laboratory air pressure, and re-planning of cleaning directions. The modifications were completed within 48 hours, including hydropower transformation; fresh air commissioning; installation of equipment furniture, and a laboratory information system (LIS); and preparation of experimental reagents and various protective materials. From June 24 to July 12, 2020, the laboratory performed a total of 41,897 nucleic acid tests for SARS-CoV-2, with a maximum of 4,030 tests in a single day. The average test turn-around time (TAT) was 7 hours from initial patient swab. Here, we summarize the aspects of laboratory reconstruction and testing process optimization to provide a multi-department cooperation model for COVID-19 testing and screening in large cities like Beijing. Our experience will provide a basis for increasing nucleic acid testing capacity in the continued fight against the COVID-19 pandemic.

The layout of the SARS-CoV-2 nucleic acid testing laboratory has an important impact on the testing workflow and on laboratory biosafety [6]. Figure S1 presents a schematic diagram of the new laboratory. The PCR laboratory generally consists of three areas: areas for reagent storage, specimen preparation, and nucleic acid amplification. Referring to experiences regarding the prevention and control of SARS and other serious infectious diseases, as well as the high-risk situation in the laboratory, we have formulated different levels of biosafety protection requirements to protect staff members from infection [7, 8]. Tables S1 and S2 list the equipment, personnel allocation, and biosafety protection requirements of our laboratory. The reagent preparation area is mainly used for the storage, preparation, and dispensing of reagents for nucleic acid testing. The nucleic acid extraction area is the area where samples are preserved and stored, nucleic acid extraction is performed, and templates are added to the amplification reaction tube. The amplification area is used for nucleic acid amplification and amplification product analysis. The instrument used for SARS-CoV-2 testing should be calibrated before use. According to guidance from the China National Accreditation Service (CNAS-GL039), after all laboratory instruments are in place, performance verification should be performed immediately [9]. The precision and accuracy of testing kits were verified, and the results obtained with randomized, blinded, negative and positive samples from another laboratory were acceptable.

To satisfy the urgent need of actively screening a large number of samples in high-risk areas in Beijing, our laboratory has optimized the number of tests performed in a given day. The following laboratory plan is determined based on a daily output of 4000 tests. Taking 93 clinical samples as a batch, 44 batches of PCR detection (4000/93 ≈ 44) are required. With 5 nucleic acid extractors and 10 PCR machines in the laboratory, 4.5 rounds of PCR tests are therefore required. The time required for each batch of nucleic acid extraction is 25 minutes. Each batch of PCR detection takes approximately 2 hours; therefore, it takes 8–10 hours to complete the 4000 tests. Figure 1 presents the actual workflow chart for this laboratory.

**Figure 1.**
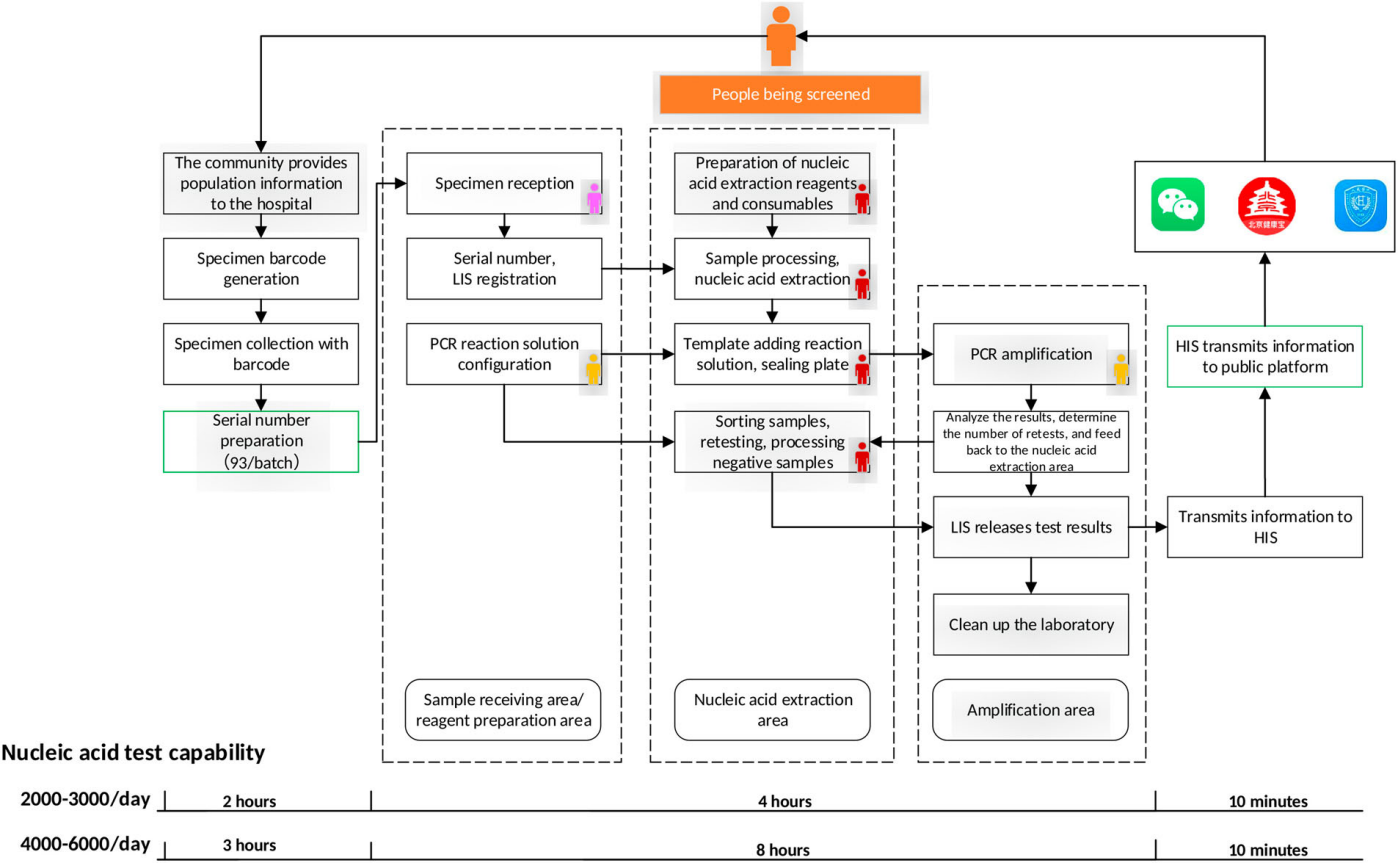
COVID-19 laboratory workflow chart in Peking University People’s Hospital (PKUPH). The bottom two rows represent the time spent in the whole process on the basis of the daily quantity of tests performed: 2000–3000 and 4000–6000, respectively. The orange figure represents the person being screened. The dashed box indicates the testing process of the nucleic acid testing laboratory, including specimen reception, reagent preparation, nucleic acid extraction, gene amplification, and release of results. The green frame represents that the specimen has been matched to a serial number from the batch of 93 samples before entering the laboratory, reducing the time required to receive specimens in the laboratory. Once the testing results were released, the hospital’s health information system platform quickly released the results on public platforms including Wechat, Beijing Jiankangbao, and PKUPH, making real-time test results available via cell phone.

Prior to nucleic acid testing, the hospital requires all identifying information from the different communities in advance and generates sample barcodes in batches. Furthermore, during the release of test results, a closed-loop informatization is created, whereby the hospital health information system (HIS) releases the results to socialized public platforms, including the WeChat, Beijing Jiankangbao, and Peking University People’s Hospital (PKUPH) applications, via cell phones. Patients and government authorities will receive nucleic acid test results as soon as possible, especially positive test results.

COVID-19 laboratory personnel must be qualified for PCR testing and interpretation. The SARS-CoV-2 Nucleic Acid Testing Laboratory maintained a streamlined operation with 7 staff members (as shown in Figure S1). One staff member each is assigned to the sample registration, reagent preparation, and amplification areas, and these three personnel can cooperate according to specific scenarios. The nucleic acid extraction area is equipped with four staff members. Of these personnel, three are responsible for adding samples to the lysate plate, and one is responsible for the operation of the nucleic acid extractor and adding templates to the system. When the number of specimens was relatively large (i.e. >3000 tests per day), the entire group made all possible efforts to keep more than 8–10 PCR machines in operation simultaneously. When a large quantity of samples require tests results quickly, the staff members in the nucleic acid extraction area enter the scanning area first.

In the reagent preparation area, the total sample quantity should be estimated as accurately as possible, and enough 96-well reaction plates should be prepared at one time to avoid a delay due to repeated preparation of reagents. The scanned and numbered specimens are then transferred to the nucleic acid extraction area through the transfer window. Sample addition should always be performed in a biosafety cabinet. A high throughput extractor was used with 96 samples per batch. To this end, the operator’s outer gloves must be taken off within the safety cabinet after disinfection. Once the sample nucleic acids have been added to the corresponding reaction tube or 96-well reaction plate, they should be covered with film and sealed within the PCR reaction tube or reaction plate. In the amplification area, a dedicated staff member removed the prepared PCR test tube, centrifuged it, set the amplification parameters according to the kit instructions, and analyzed the results. Due to the technical characteristics of the sensitivity and limitations of testing, two staff members reviewed the test results comprehensively. The analysis results are recorded in real time, and the codes of the specimens that require re-examination are recorded on the re-examination record sheet. After the results are released from the laboratory, the hospital’s HIS platform quickly releases the results on the public application platforms of PKUPH, WeChat, and Beijing Jiankangbao within the same day. Those who have been tested can also check their test reports through a mobile phone application.

The quality control of nucleic acid testing is reflected in all elements, including the pre-analysis, analysis and post-analysis stage. Specimen collection personnel are trained according to guidelines on the collection of oropharyngeal and nasopharyngeal swabs during large-sample screening [4]. Laboratory staff should fully understand the work process and operation. External quality assessment should per performed regularly. A double-signature system is implemented, whereby the report is reviewed and approved by two different staff members. The LIS can ensure the release of each batch’s results.

The rapid and successful control of the recent COVID-19 epidemic in Beijing was mainly attributable to the strengthening of laboratory SARS-CoV-2 nucleic acid testing capacity. Optimization of workflow of the whole testing process and shortening the time spent for each step are key to managing the large testing volumes in an infectious disease epidemic.

## Supplementary Material

03_Supplement.docx

## References

[1] Guan WJ, Ni ZY, Hu Y, et al. Clinical characteristics of coronavirus disease 2019 in China. N Engl J Med. 2020;382:1708–1720. 32109013 10.1056/NEJMoa2002032 PMC7092819

[2] Tang YW, Schmitz JE, Persing DH, et al. Laboratory diagnosis of covid-19: current issues and challenges. J Clin Microbiol. 2020;26; 58(6):e00512-20. 10.1128/JCM.00512-20 PMC7269383 32245835

[3] Li Z, Chen Q, Feng L, et al. Active case finding with case management: the key to tackling the covid-19 pandemic. Lancet. 2020;396:63–70. 32505220 10.1016/S0140-6736(20)31278-2 PMC7272157

[4] World health organization. Laboratory testing for coronavirus disease 2019 (covid-19) in suspected human cases: interim guidance. [cited 2 Mar 2020]. https://apps.Who.Int/iris/bitstream/handle/10665/331329/who-covid-19-laboratory-2020.4-eng.Pdf.

[5] http://health.people.com.cn/n1/2020/0624/c14739-31758917.html.

[6] Mifflin TE. Setting up a pcr laboratory. CSH protocols. 2007. doi:10.1101/pdb.top14. 21357132

[7] Htun H, Lim D, Kyaw W, et al. Responding to the covid-19 outbreak in Singapore: staff protection and staff temperature and sickness surveillance systems. Clin Infect Dis. 2020;21:ciaa468. 10.1093/cid/ciaa468 PMC7188160 32315026

[8] MacIntyre C, Wang Q. Physical distancing, face masks, and eye protection for prevention of covid-19. Lancet. 2020;395:1950–1951. 32497511 10.1016/S0140-6736(20)31183-1 PMC7263820

[9] Guidance on the performance verification for molecular diagnostic procedure. China national accreditation service for conformity assessment. https://www.cnas.org.cn/images/rkgf/sysrk/rkzn/2019/04/04/ee941bdabea79c7d6d9128a28852297c.Pdf.

