# Supplementary material for: Increasing SARS-CoV-2 nucleic acid testing capacity during the COVID-19 epidemic in Beijing: experience from a general hospital
Source: Emerg Microbes Infect. 2020 Nov 1;9(1):2358–60. doi: 10.1080/22221751.2020.1837016 (PMC7605315; doi:10.1080/22221751.2020.1837016)

**Table S1. Levels of biosafety protection required in Peking University People’s Hospital (PKUPH)**

| Protection level | Specific requirements |
| --- | --- |
| Level 1 | Medical surgical masks, latex gloves, work clothes, hand hygiene, medical protective caps can be worn |
| Level 2 | Medical protective masks or N95 masks, latex gloves, gowns over work clothes, medical protective caps, and hand hygiene. Goggles can be added when necessary (e.g., when there is a risk of splashing) |
| Level 3 | Medical protective mask or N95, single or double-layer medical protective cap, face screen, goggles, double-layer latex gloves (conditions permit, different colors), protective clothing over work clothes, shoe covers, and hand hygiene. Double-layer masks when necessary (i.e., outer medical surgical mask, inner N95 or medical protective mask) |
| Special protection | In cases in which the patient coughs severely and does not have a respiratory barrier, three-level protection should be adopted including double-layer protective clothing or protective clothing plus isolation gown, double-layer mask (i.e., outer medical surgical mask, inner N95 or medical protective mask), comprehensive respiratory protection device, three-layer latex gloves, double-layer medical protective cap, etc. |

**Table S2. Equipment and personnel protection in the COVID-19 nucleic acid testing laboratory of Peking University People’s Hospital (PKUPH)**

| Room | Instrument (Quantity) | Number of Staff | Biosafety Protection Level |
| --- | --- | --- | --- |
| Reagent Preparation Room | -20℃ Refrigerator (2), 4-6℃ Refrigerator (1), Centrifuge (1), Vortex Mixer (1), Calculator (1), Mobile UV lamp (1), Walkie-Talkie (1) | 1 | Level 2 Plus |
| Sample Preparation Room | Biological Safety Cabin (2), Nucleic Acid Extractor (5), Centrifuge (2), Water Bath (1), Mobile UV Lamp (1), Walkie-Talkie (1) | 4 | Level 3 |
| Sample Reception | Computer (2), 4-6℃ Refrigerator (2), Mobile UV lamp (1), Walkie-Talkie (1) | 1 | Level 2 Plus |
| Amplification Room | PCR Machine (10), Centrifuge (2), Uninterrupted Power Supply, UPS (2), -20℃ Refrigerator (2), Computer (10), Mobile UV Lamp (1 set), Walkie-Talkie (1 set) | 1 | Level 2 Plus |
| Autoclave Room | Autoclave (3) | # | Level 2 Plus |

# The work in the autoclave room can be coordinated by the staff in the amplification area, reagent preparation area, or specimen reception area.

**Figure S1. Schematic diagram of the COVID-19 nucleic acid testing laboratory**

Figure legend: The green arrows indicate the flow of personnel in the laboratory, and the red arrows indicate the flow of specimens in the laboratory. The personnel represented in red are those in the nucleic acid extraction area. Once these personnel enter the nucleic acid extraction area, they will not be allowed to enter other areas of the laboratory. The personnel represented in pink are those in the specimen receiving area, responsible for receiving specimens and the entry of detection information in the laboratory information system. The personnel represented in yellow are those in the reagent preparation and amplification areas, responsible for the preparation of the PCR reaction solution, PCR amplification, and product analysis.


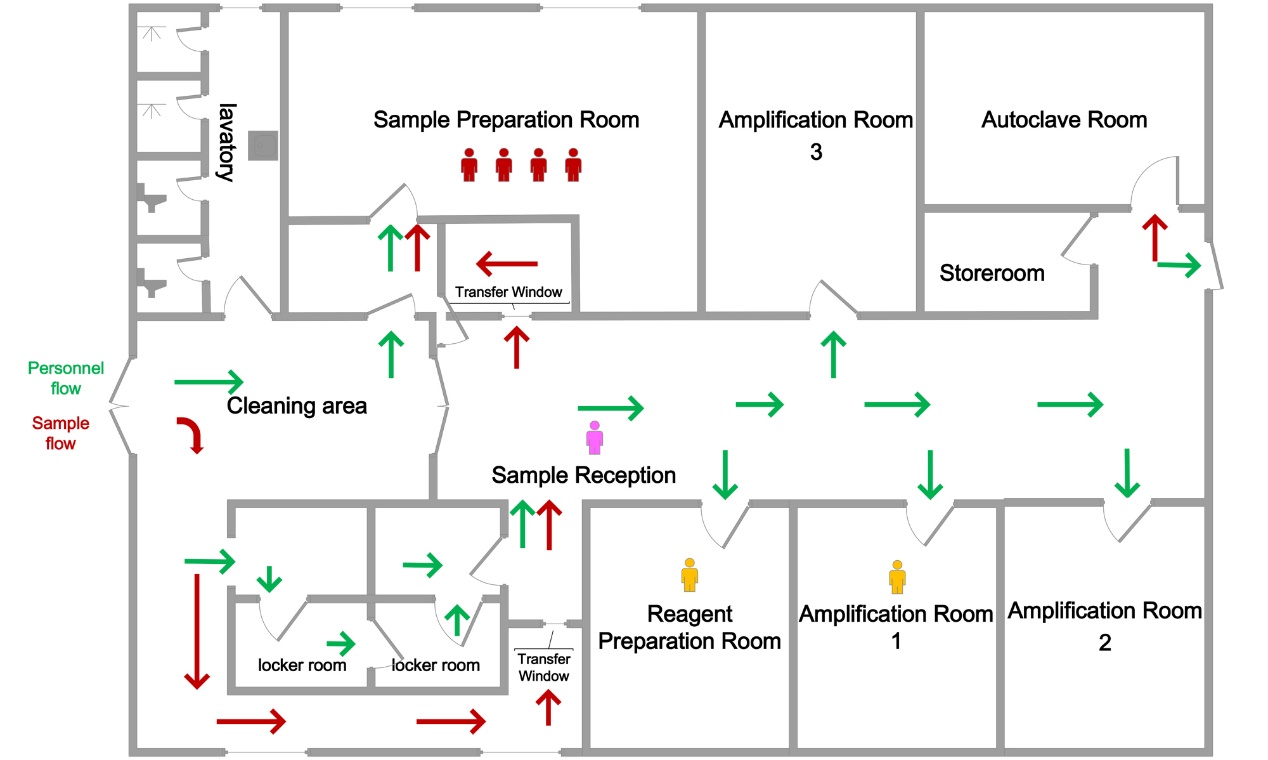

Supplement: 03_Supplement.docx [file TEMI_A_1837016_SM3693.docx]
